# Early Treatment Critical: Bexarotene Reduces Amyloid-Beta Burden In Silico

**DOI:** 10.1371/journal.pone.0153150

**Published:** 2016-04-13

**Authors:** Joseph Rosenthal, Georges Belfort, David Isaacson

**Affiliations:** 1 Department of Mathematical Sciences, Rensselaer Polytechnic Institute, Troy, New York, United States of America; 2 Howard P. Isermann Department of Chemical and Biological Engineering and Center for Biotechnology and Interdisciplinary Studies, Rensselaer Polytechnic Institute, Troy, New York, United States of America; UC Santa Barbara, UNITED STATES

## Abstract

Amyloid-beta peptides have long been implicated in the pathology of Alzheimer’s disease. Bexarotene, a drug approved by the U.S. Food and Drug Administration for treating a class of non-Hodgkin’s lymphoma, has been reported to facilitate the removal of amyloid-beta. We have developed a mathematical model to explore the efficacy of bexarotene treatment in reducing amyloid-beta load, and simulate amyloid-beta production throughout the lifespan of diseased mice. Both aspects of the model are based on and consistent with previous experimental results. Beyond what is known empirically, our model shows that low dosages of bexarotene are unable to reverse symptoms in diseased mice, but dosages at and above an age-dependent critical concentration can recover healthy brain cells. Further, early treatment was shown to have significantly improved efficacy versus treatment in older mice. Relevance with respect to bexarotene-based amyloid-beta-clearance mechanism and direct treatment for Alzheimer’s disease is emphasized.

## Introduction

In 2012, Cramer et al. repurposed a lymphoma drug, bexarotene, to be used as a potentially disease modifying treatment for Alzheimer’s disease (AD) [1]. The group treated diseased mice with the drug and found an increase in cognitive function and a decrease in amyloid-beta (Aβ), one of the hallmark proteins of AD. The purported recovery from AD in a mouse model with the addition of a retinoid X receptor agonist, bexarotene, that overproduced Apolipoprotein E (ApoE) and led to the eventual removal of Aβ from the brain, demonstrated that effective downstream clearance could be critical for the reversal of AD in a mouse.

Other groups have attempted to replicate the results of Cramer et al. to varying degrees of success. Many of the dissenting studies used generic bexarotene with a variety of formulations [2–5]. Cramer et al. made use of the micronized and capsuled Targretin, which has improved efficacy over generic bexarotene [1, 6]. Veeraghavalu and Zhang mimicked the conditions of the original experiment by Cramer et al., but were unable to show that bexarotene had a significant effect on plaque deposition [7]. Boehm-Cagan et al. showed that bexarotene can also modify apoE4-based neuronal decline and apoE4-associated tau hyperphosphorylation in *apoE4* mice [8].

Beyond animal models, some studies have been performed on AD patients: Pierrot et al. [9] found that six months of 300 mg of daily Targretin treatment can increase memory in human AD patients by up to 40%, while also decreasing the concentration of tau in cerebrospinal fluid. Cummings et al. [10] showed that for patients with mild-to-moderate AD, bexarotene was able to reduce brain Aβ_42_ levels in noncarriers of *apoE4*, though they demonstrated that the drug was unable to reduce Aβ_42_ in *apoE4* carriers.

The goal of this paper is to present the simplest mathematical model that describes the production of Aβ and the treatment of AD mice with a RXR agonist while being consistent with the experimental work of Trinchese and Liu [11] and Cramer et al. [1]. For this model, we are specifically considering bexarotene treatment with the micronized Targretin, but this work can be generalized to apply to any RXR agonist. The effects of variation in bexarotene dosage frequency, and also the effect of bexarotene when mouse age is varied, will be demonstrated. We describe the construction of our model in, and in Constructing the model we present the results of the simulation and comparisons of our results to experimental data from Trinchese and Liu [11], Cramer et al. [1], and Veeraghavalu and Zhang [7]. Finally, our conclusion is given in Comparison of healthy brain cells and plaque.

## Model

### Constructing the model

Note that each function, rate constant and parameter introduced in this section is also listed in Tables 1 and 2

**Table 1:** Index of function definitions.

Variable	Unit	Definition
*N*	vol.^−1^	Number of healthy brain cells (neurons and glial cells) per volume.
*N*_*D*_	vol.^−1^	Number of diseased brain cells per volume.
*A*_*pp*_	vol.^−1^	Number of amyloid precursor proteins per volume.
*S*_*γ*_	vol.^−1^	Number of gamma-secretase complexes per volume.
*A*	pmol ⋅ mg^−1^	Amount of amyloid beta per mass of protein.
*R*	vol.^−1^	Number of retinoid X receptors per volume.
*P*_0_	vol.^−1^	Number of Apolipoprotein E (ApoE) peptides oer volume.
*B*	mg ⋅ kg^−1^	Mass of Bexarotene per mass of subject.

**Table 2:** Index of rate constant and parameter definitions.

Variable	Unit	Definition
*k*_*d*_	day^−1^	Rate at which healthy brain cells convert to diseased brain cells.
*k*_*h*_	day^−1^	Rate at which diseased brain cells convert to healthy brain cells.
*λ*_*d*_	day^−1^	Maximum rate of healthy brain cells converting to diseased brain cells.
*μ*_*d*_	day^−1^	Rate of diseased brain cell death.
*k*_*γ*_	pmol ⋅ (mg ⋅ day ⋅ vol.^2^)^−1^	Rate at which gamma-secretase forms Aβ_42_.
*k*_*A*_	pmol ⋅ mg^−1^ day^−1^	Rate at which Aβ_42_ is produced.
*k*_*R*_	kg ⋅ mg^−1^ ⋅ day^−1^	Rate at which bexarotene binds with RXR.
*k*_*B*_	kg ⋅ mg^−1^ ⋅ day^−1^	Rate at which ApoE is produced due to bexarotene.
*k*_*R*_	day^−1^	Rate at which ApoE is naturally produced by RXR agonization.
*k*_*P*_0__	vol. ⋅ hr.^−1^	Rate at which unbound ApoE binds to an Aβ oligomer.
*ρ*_*pp*_	—	Number of amyloid precursor proteins per healthy brain cell
*ρ*_*pp*′_	—	Number of amyloid precursor proteins per diseased brain cell
*ρ*_*γ*_	—	Number of γ-secretase complexes per healthy brain cell
*ρ*′_*γ*_	—	Number of γ-secretase complexes per diseased brain cell
*ρ*_*R*_	—	Number of RXR receptors per brain cell
*t*_*B*_	days	Lag time to introduction of bexarotene.
*B*_0_	mg ⋅ kg^−1^	Initial mass of bexarotene per mass of subject.
*r*	day^−1^	Bexarotene rate constant.
*L*	days	Period of bexarotene increase.
*α*_*d*_	pmol ⋅ mg^−1^	Equilibrium constant.

Let *N* be the concentration of healthy brain cells, that is, healthy neurons and glial cells. Let *N*_*d*_ represent the concentration of diseased brain cells. Assume that both concentrations are the number of cells in a volume with an initial count of 100 healthy brain cells. Assume that healthy brain cells become diseased at a rate of *k*_*d*_(*A*), with a reverse rate constant of *k*_*h*_(*A*), where *A* gives the concentration of Aβ_42_. Assume that diseased brain cells become damaged cells with rate constant *μ*_*d*_, so that

$$N\overset{k_{d}(A)}{\underset{k_{h}(A)}{⇌}}\;N_{d}\;\overset{\mu_{d}}{\to }\text{ damaged cell.}$$ (1)

From Eq 1 we obtain the ordinary differential equations corresponding to the concentration of healthy and diseased brain cells:

$$\begin{array}{c} \frac{dN}{dt}=-k_{d}\left(A\right)N+k_{h}\left(A\right)N_{d} \end{array}$$ (2)

$$\begin{array}{c} \frac{dN_{D}}{dt}=k_{d}\left(A\right)N-k_{h}\left(A\right)N_{D}-\mu_{d}N_{d}. \end{array}$$ (3)

We assume Michaelis-Menten kinetics with the rate constants *λ*_*d*_, *α*_*d*_ for *k*_*d*_ and *k*_*h*_ [12, 13]:

$$\begin{array}{c} k_{d}\left(A\right)=\frac{\lambda_{d}A}{A+\alpha_{d}}, \end{array}$$ (4)

$$\begin{array}{c} k_{h}\left(A\right)=\lambda_{d}-k_{d}\left(A\right). \end{array}$$ (5)

While APP is cleaved by both β and γ-secretases, in this model it is assumed that the formation of Aβ_42_ is rate-limited by γ-secrease and thus the lumped rate constant *k*_*γ*_ is used for both cleavage events. Similarly, it is assumed that this *k*_*γ*_ also accounts for the time required for Aβ_42_ to oligermerize. Let the concentration of secretases required to cleave the amyloid precursor protein be given by *S*_*γ*_, so that

$$A_{pp}\;+\;S_{\gamma }\;\overset{k_{\gamma }}{\to }\;A.$$ (6)

Aβ_42_ binds to ApoE at a rate of *k*_*P*_0__, and is removed across the blood-brain barrier from the system:

$$A\;+\;P_{0}\;\overset{k_{p_{0}}}{\to }\text{ removal of A}\beta_{42},$$ (7)

and thus by Eqs 6 and 7 we have that

$$\begin{array}{c} \frac{dA}{dt}=k_{\gamma }A_{pp}S_{\gamma }-k_{P_{0}}AP_{0}. \end{array}$$ (8)

Note that *P*_0_ only represents the concentration of ApoE produced due to bexarotene interaction with RXR; background concentrations of ApoE are not considered for this model.

It is assumed that the production of APP is significantly faster than the loss of APP due to Aβ_42_ production. Further, assume that the concentration of APP is proportional to both the concentration of healthy and diseased brain cells, so that

$$\begin{array}{c} A_{pp}=\rho_{pp}N+\rho_{pp^{\prime }}N_{d}, \end{array}$$ (9)

where *ρ*_*pp*_ and *ρ*_*pp*′_ are the concentrations of *APP* per healthy brain cells and diseased brain cells, respectively.

Assume that the concentration of secretase complexes is proportional to the concentration of healthy brain cells and diseased brain cells, so that

$$\begin{array}{c} S_{\gamma }=\rho_{\gamma }N+\rho_{\gamma^{\prime }}N_{d}, \end{array}$$ (10)

where *ρ*_*γ*_ and *ρ*_*γ*′_ are the number of γ-secretase complexes per healthy brain cell and diseased cell, respectively. Note that it is assumed that γ-secretase is not lost when the APP cleavage event occurs.

It follows from Eqs 9 and 10 that

$$\begin{array}{c} A_{pp}S_{\gamma }=\rho_{\gamma }\rho_{pp}N^{2}+\left(\rho_{\gamma }\rho_{pp^{\prime }}+\rho_{\gamma^{\prime }}\rho_{pp}\right)NN_{d}+\rho_{\gamma^{\prime }}\rho_{pp^{\prime }}{N_{d}}^{2}. \end{array}$$ (11)

It has been reported that neuronal injury leads to the upregulation of APP [14, 15]; fitting to Trinchese and Liu [11] yielded values of *ρ*_*pp*_ that are several orders of magnitude less than that of *ρ*_*pp*′_, and thus we assume that

$$\begin{array}{c} \rho_{pp}=0. \end{array}$$ (12)

We also assume that

$$\begin{array}{c} \rho_{\gamma }=\rho_{\gamma^{\prime }}, \end{array}$$ (13)

and thus we have that

$$\begin{array}{c} \frac{dA}{dt}=k_{A}\left(N_{d}^{2}+N_{d}N\right)-k_{P_{0}}AP_{0}, \end{array}$$ (14)

where *k*_*A*_ = *k*_*γ*_ *ρ*_*pp*′_2*ρ*_*γ*_.

Let *B* represent the concentration of bexarotene (mg ⋅ kg^−1^), define *R* as the concentration of RXR (number per volume), and let *P*_0_ represent the concentration of unbound ApoE (number of peptides per volume). Bexarotene binds to RXR to promote the production of ApoE at a rate of *k*_*R*_, and we assume that the unbinding rate of bexarotene from *RXR* is lumped into *k*_*R*_, thus yielding the following reaction:

$$B\;+\;R\;\overset{k_{B}}{\to }\;P_{0}.$$ (15)

Note that our reaction scheme is given in Table 3.

**Table 3:** Reaction scheme of Aβ production and treatment.

Description	Reaction scheme
Conversion of healthy brain cells to and from diseased cells and eventual permanent neuronal damage	$N\overset{k_{d}\;\left(A\right)}{\underset{k_{h}\;\left(A\right)}{⇌}}N_{d}\overset{\mu_{d}}{\to }\text{damaged cell}$
APP cleavage event	$A_{pp}\;+\;S_{\gamma }\;\overset{k_{\gamma }}{\to }\;A$
PPAR: γ and LXR:RXR agonization and ApoE production	$B\;+R\;\overset{k_{B}}{\to }\;P_{0}$
ApoE-Aβ binding event	$A\;+\;P_{0}\;\overset{kP_{0}}{\to }\;removal\;of\;A\beta_{42}$

From Eqs 7 and 15 we can write the differential equation

$$\begin{array}{c} \frac{dP_{0}}{dt}=k_{R}RB-k_{P_{0}}AP_{0}. \end{array}$$ (16)

It is assumed that when bexarotene binds to RXR, RXR is not removed from the system, and we assume that the concentration of RXR is proportional to the concentration of healthy brain cells:

$$\begin{array}{c} R=\rho_{R}N. \end{array}$$ (17)

Put *k*_*B*_ = *k*_*R*_*ρ*_*R*_ and Eq (16) simplifies to

$$\begin{array}{c} \frac{dP_{0}}{dt}=k_{B}NB-k_{P_{0}}AP_{0}. \end{array}$$ (18)

**Fig 1:**
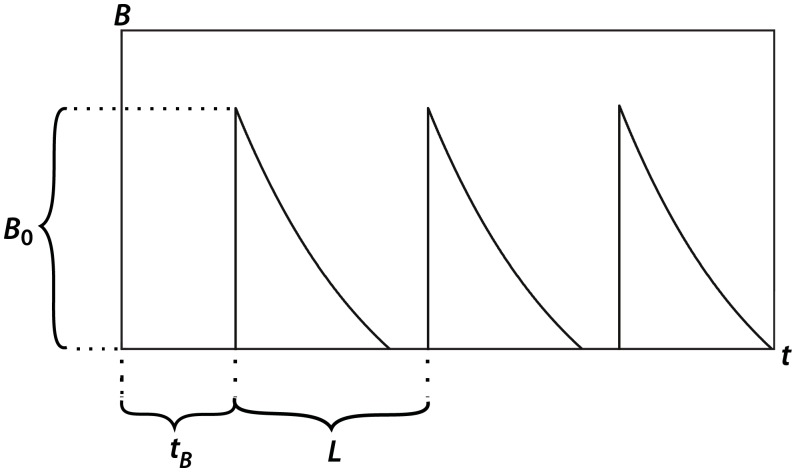
Generalized bexarotene concentration as a function of time.

The entire system is then described by the following ordinary differential equations:

$$\begin{array}{c} \frac{dN}{dt}=-k_{d}\left(A\right)N+k_{h}\left(A\right)N_{d}, \end{array}$$ (19)

$$\begin{array}{c} \frac{dN_{D}}{dt}=k_{d}\left(A\right)N-k_{h}\left(A\right)N_{D}-\mu_{d}N_{d}, \end{array}$$ (20)

$$\begin{array}{c} \frac{dA}{dt}=k_{A}\left(N_{d}^{2}+N_{d}N\right)-k_{P_{0}}AP_{0}, \end{array}$$ (21)

$$\begin{array}{c} \frac{dP_{0}}{dt}=k_{B}NB-k_{P_{0}}AP_{0}, \end{array}$$ (22)

where we let *B*(*t*), shown in Fig 1, represent the concentration (mg ⋅ kg^−1^) of bexarotene in the system with respect to time:

$$\begin{array}{c} B\left(t\right)=B_{0}\exp\left[rL\left(\left\lfloor \frac{t-t_{B}}{L}\right\rfloor -\frac{t-t_{B}}{L}\right)\right]\cdot \chi_{\left[t_{B},\infty \right)}\left(t\right), \end{array}$$ (23)

where *B*_0_ represents the concentration of bexarotene in a given dosage (mg ⋅ kg^−1^); *t*_*B*_ gives the delay before treatment is started (days), and *L* represents the period between dosages (days). The constant *r* (day^−1^) is chosen based on the half-life of bexarotene. We then define the following:

$$\begin{array}{c} \left\lfloor \frac{t-t_{B}}{L}\right\rfloor =\max\left\{n\in \mathbb{Z}\;|\;n\leq \frac{t-t_{B}}{L}\right\}, \end{array}$$ (24)

$$\begin{array}{c} \chi_{\left[t_{B},\infty \right)}\left(t\right)=\left\{\begin{array}{cc} 1 & \text{if}\;t\in [t_{B},\infty ),\\ 0 & \text{otherwise.} \end{array}\right. \end{array}$$ (25)

## Methods

The parameters *λ*_*d*_, *α*_*d*_, *μ*_*d*_, and *k*_*A*_ were first fit to Aβ_42_ load data from Trinchese and Liu [11]. The Aβ_42_ load data given by Trinchese and Liu [11] appeared to increase rapidly after a short lag period of approximately 3.5 months, and so *α*_*d*_ was chosen to reflect this. In order to find *λ*_*d*_ and *k*_*A*_, the parameter space was explored. The values of *A*(*t*) for each time *t* reported in Trinchese and Liu [11] were recorded and compared to the corresponding experimental result. The difference between the computed and experimental result was aggregated over each time, and the square of this difference was minimized.

The remaining parameters *k*_*P*_0__ and *k*_*B*_ were then fit to percent decreases in soluble Aβ_42_ given by Cramer et al. [1]. Percent changes in simulated Aβ_42_ load were calculated as shown in Eq 26 and subtracted from that of Cramer et al. [1]. The square of each difference was aggregated, and the sum was reduced while exploring the parameter space.

The parameters used are given in Table 4, and the sensitivity of the system to perturbations in the parameters is discussed in Supporting Information.

**Table 4:** Initial values and parameters used for simulation plots. The value for *A*(0) was obtained from Fig 3 of Trinchese and Liu [11]. The value of *r* was calculated using bexarotene half-life data from Fig 1 of Landreth and Cramer [6].

Parameter	Value
*N* (0)	100 vol.^−1^
*N*_*d*_ (0)	0 vol.^−1^
*A* (0)	0.25 pmol ⋅ mg^−1^
*P*_0_ (0)	0 vol.^−1^
*λ*_*d*_	6.1 ⋅ 10^−2^ day^−1^
*α*_*d*_	17 vol.^−1^
*μ*_*d*_	5 ⋅ 10^−3^ day^−1^
*k*_*A*_	3.5 ⋅ 10^−4^ pmol ⋅ (mg ⋅ day ⋅ vol.^2^)^−1^
*k*_*P*_0__	4.4 ⋅ 10^−2^ vol. ⋅ day
*k*_*B*_	5 ⋅ 10^−2^ kg ⋅ mg^−1^ day^−1^
*r*	15.26 day^−1^

## Results

### Untreated *APP*/*PS1* mice

**Fig 2:**
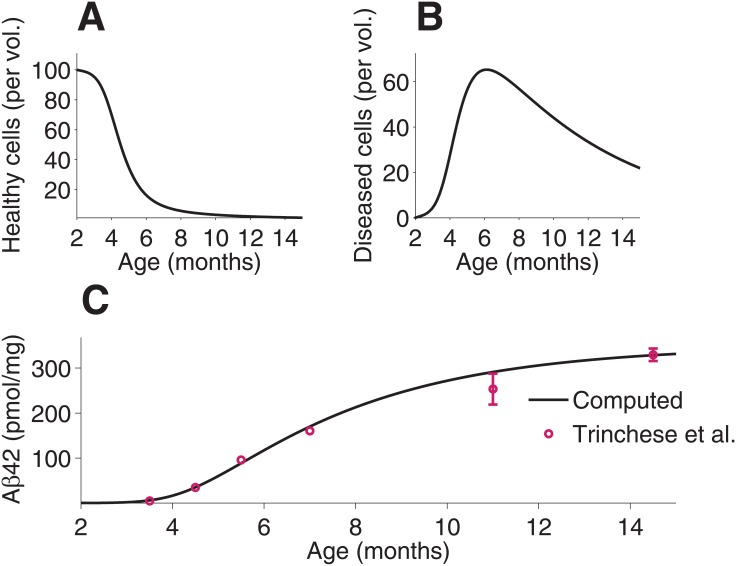
Simulation of *APP*/*PS1* mice from two-months-old to 15-months-old with no treatment. The concentration of healthy brain cells (**A**) and diseased brain cells (**B**) with respect to time (**C**) are given. The computed A*β*_42_ load is presented and compare to experimental data given in Fig 3 in Trinchese and Liu [11].

A two-month-old mouse is simulated until 15-months-old for comparison to the experimental results of Trinchese and Liu. The concentration of healthy brain cells (Fig 2A) decreases monotonically, while the concentration of diseased brain cells (Fig 2B) increases until the cells become damaged. The concentration of Aβ_42_ (Fig 2C) increases sigmoidally with respect to time. A very close approximate fit to the results of Trinchese and Liu is demonstrated, and the simulation falls within the reported margin of error of the results given by Trinchese and Liu [11].

### *APP*/*PS1* mice with daily treatment

**Fig 3:**
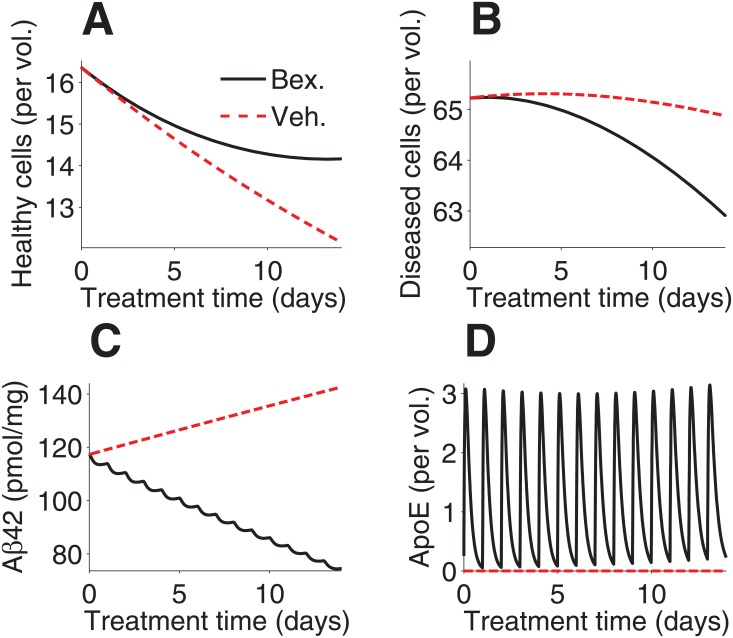
Simulation of six-month-old *APP*/*PS1* mice with treatment. (**A**) 14-day simulations of healthy brain cells, (**B**) diseased brain cells, (**C**) Aβ_42_ load, and (**D**) ApoE in six-month-old *APP*/*PS1* mice, are shown for mice given no treatment and those given 100 mg ⋅ kg^−1^ bexarotene treatment.

In order to compare our model to the experimental results by Cramer et al. [1], the following simulations were run: a six-month-old *APP*/*PS1* mice with three, seven, and 14 days of treatment (Fig 3); a simulation of a nine-month-old *APP*/*PS1* mouse with 90 days of treatment (S1 and S2 Figs); and a simulation of an 11-month-old *APP*/*PS1* mouse with seven days of treatment (S3 Fig).

Six-month-old *APP*/*PS1* mice showed a decrease in the concentration of both healthy and diseased brain cells with 14 days of treatment (Fig 3A and 3B), though the rates of both are slower than in the case without treatment. A significant decrease of Aβ_42_ load is evident with treatment, while without treatment, it increases steadily (Fig 3C). ApoE is seen to increase with each dose of bexarotene (Fig 3D).

**Fig 4:**
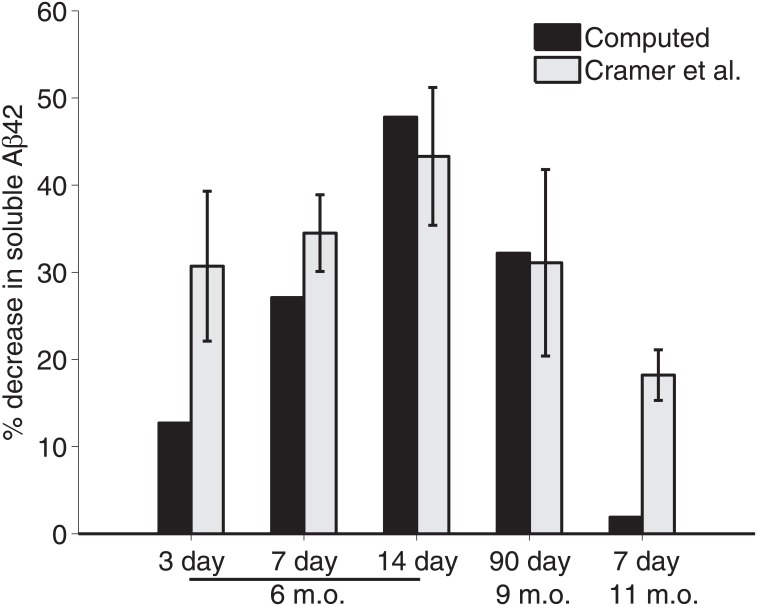
Comparison of computed data from the model and experimental data from Cramer et al. (A) Computed data from the model is compared to that from Fig 2 of Cramer et al. [1], S4 and S5 Figs of the supporting online materials to Cramer et al. [1] of *APP*/*PS1* mice at six-months-old given treatment for three, seven and 14 days; nine-months-old given treatment for 90 days; and at 11-months-old given seven days of treatment. All treatment is for 100 mg ⋅ kg^−1^ bexarotene.

**Fig 5:**
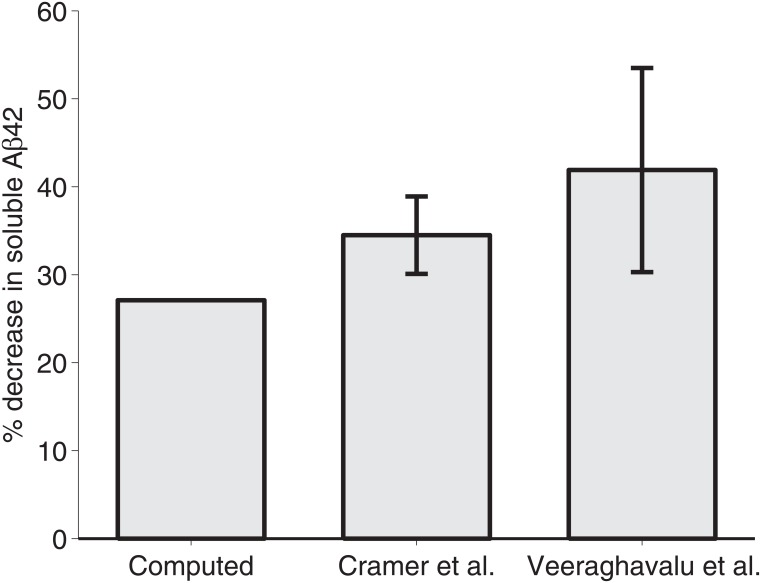
Comparison of computed data from the model and experimental data from Cramer et al. and Veeraghavalu et al. Computed data of six-month-old *APP*/*PS1* mice treated with 100 mg ⋅ kg^−1^ of bexarotene for seven days is compared to the experimental results of Cramer et al. [1] and the results presented in Fig 1 of Veeraghavalu et al. [7].

In Fig 4, computed values from the model are shown to approximate the trend of decrease in soluble Aβ_42_ given by Cramer et al. [1], with the closest approximations being for younger mice with longer treatments. In the simulation of six-month-old *APP*/*PS1* mice given seven days of treatment, the computed values closely approximate the experimental results of Cramer et al. [1] and fall within the margin of error of those given by Veeraghavalu et al. [7] (Fig 5).

Note that the percent change demonstrated in Figs 4 and 5 is defined by the following:

$$\begin{array}{c} \%\Delta_{\text{comp}}=\frac{A_{0}^{\text{comp}}\left(t_{f}\right)-A_{100}^{\text{comp}}\left(t_{f}\right)}{A_{0}^{\text{comp}}\left(t_{f}\right)}, \end{array}$$ (26)

where $A_{B_{0}}^{\text{comp}}\left(t_{f}\right)$ represents the computed value of *A* at the end of a treatment (*t*_*f*_) with *B*_0_ mg ⋅ kg^−1^ of bexarotene.

### *APP*/*PS1* mice with varying treatment frequency and dosage

In order to explore changes in the frequency of bexarotene treatment, the dosage of bexarotene was varied from 0 mg ⋅ kg^−1^ to 1000 mg ⋅ kg^−1^ for six-month-old *APP*/*PS1* mice over 14 days of treatment.

**Fig 6:**
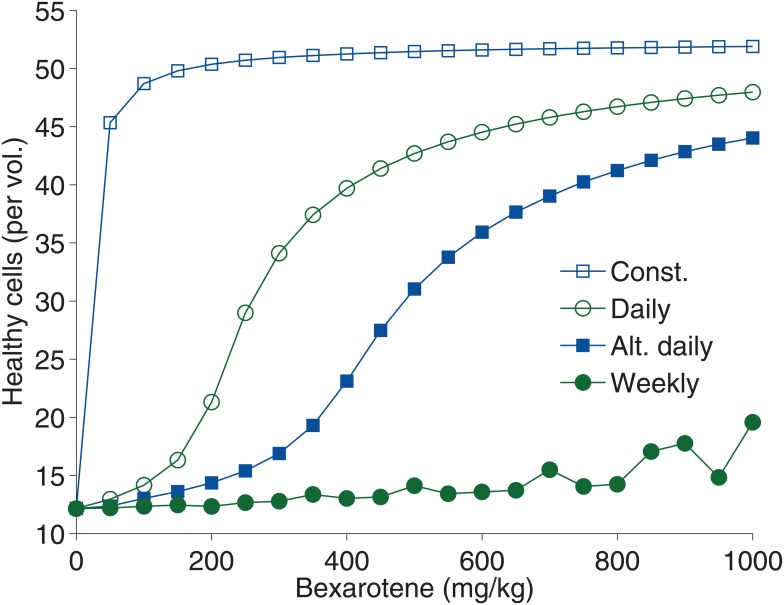
Simulation of healthy brain cell concentration for six month-old *APP* / *PS1* mouse with varying bexarotene dosage and frequency of treatment. Treatment is varied from constant, daily, alternate-day, and weekly addition of bexarotene. Bexarotene is given in dosages from 0 mg ⋅ kg^−1^ to 1000 mg ⋅ kg^−1^ of a period of two weeks.

Approximately 5 mg ⋅ kg^−1^ of constant bexarotene is shown to yield the same effect as 100 mg ⋅ kg^−1^ of daily treatment (Fig 6). Weekly treatment is much less effective, requiring nearly seven times the dosage to match the effect of the 100 mg ⋅ kg^−1^ daily treatment.

### *APP*/*PS1* mice with varied age and treatment dosage

**Fig 7:**
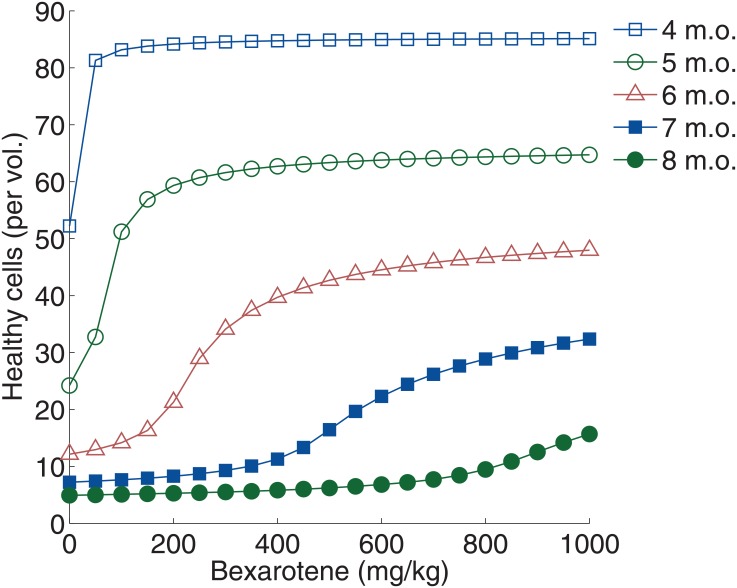
Simulation of healthy brain cell concentration with varying bexarotene dosage and age of *APP*/*PS1* mice. The concentration of healthy brain cells at the end of treatment is reported for four month-old mice, five month-old mice, six month-old mice, seven month-old mice, and eight month-old mice. Bexarotene is varied from 0 mg ⋅ kg^−1^ to 1000 mg ⋅ kg^−1^ over a period of two weeks.

Four-to-eight-month-old *APP*/*PS1* mice are simulated in order to investigate the efficacy of bexarotene throughout the progression of AD (Fig 7). Treatment dosage is varied from 0 mg ⋅ kg^−1^ to 1000 mg ⋅ kg^−1^ of daily-added bexarotene, and the mice are treated for 14 days.

The treatment of the four-month-old mouse is seen to be much more effective than that of the older mice, recovering nearly all of the remaining brain cells with less than 100 mg ⋅ kg^−1^ of daily treatment. With the older mice, Aβ burden has become too significant, and not enough healthy brain cells are available to produce ApoE, thus the bexarotene treatment is less effective. This coincides with result of Balducci et al. reported for 12-month-old mice, which showed that bexarotene was unable to reverse brain atrophy or plaque deposition in 12-month-old *APP*/*PS1* mice [16].

From Fig 7, we see that approximately 50mg ⋅ kg^−1^ is the critical dosage required to recover healthy brain cells in a four-month-old APP/PS1 mouse. For the five-month-old mice the critical dosage is approximately 150mg ⋅ kg^−1^, and at six months, the critical dosage increases to 300mg ⋅ kg^−1^. For this range, the critical dosage increases exponentially with respect to mouse age.

### Comparison of healthy brain cells and plaque

While there is no causative link between Aβ plaque and the number of brain cells, we compare the percent increase in simulated healthy brain cells to the percent decrease plaque area reported by Cramer et al. [1].

Let $N_{B_{0}}^{\text{comp}}\left(t_{f}\right)$ represent the computed concentration of total brain cells (healthy and diseased) in the cortex of an *APP*/*PS1* mouse. In order to compare the decrease in plaque area from Cramer et al. [1] to increase in the concentration of healthy brain cells, the percent change of each is calculated:

$$\begin{array}{c} \%\Delta_{\text{comp}}=\frac{N_{100}^{\text{comp}}\left(t_{f}\right)-N_{0}^{\text{comp}}\left(t_{f}\right)}{N_{0}^{\text{comp}}\left(t_{f}\right)}. \end{array}$$ (27)

**Fig 8:**
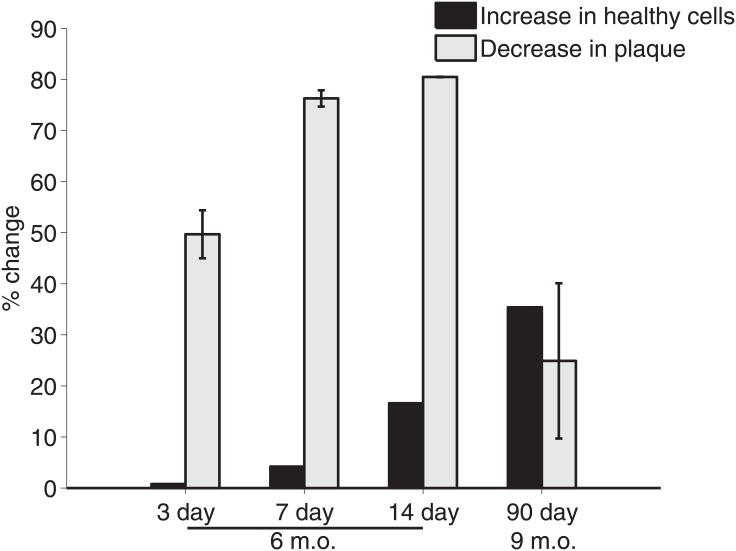
Percent increase in healthy brain cell concentration and percent decrease in Aβ plaque area. *APP*/*PS1* mice at six-months-old are given treatment for three, seven and 14 days; and nine-months-old given treatment for 90 days. The percent decrease of plaque area was estimated with cortex measurements given by Fig 2 of Cramer et al. [1] and S5 Fig of the supporting online materials to Cramer et al. [1].

Fig 8 shows a comparison of the percentage decrease in plaque area reported in Cramer et al. [1] with the percentage increase in healthy brain cells computed from this model.

## Conclusion

With our model, we are able to predict Aβ_42_ load throughout the adult life of an *APP*/*PS1* mouse and reproduce experimental results presented by Trinchese and Liu [11]. Aβ_42_ response to bexarotene in *APP*/*PS1* mice was simulated, and the model approximates the results of both Cramer et al. [1] and Veeraghavalu and Zhang [7].

An age-dependent critical dosage was found to reduce Aβ load and recover healthy brain cells in *APP*/*PS1* mice, and this critical dosage was shown to increase exponentially with respect to mouse age for six-month-old mice and younger. If treated as late as four-months-old, we have shown that under 100 mg ⋅ kg^−1^ of daily bexarotene treatment can reverse healthy brain cell damage in *APP*/*PS1* mice. Simulations of nine-month-old and 11-month-old *APP*/*PS1* mice show that bexarotene is significantly less effective at reducing Aβ_42_ load, which suggests that early treatment can have markedly improved efficacy over that in older mice.

Treatment frequency was varied, and indicated that under 5 mg ⋅ kg^−1^ of constant bexarotene treatment can have the same efficacy as 100 mg ⋅ kg^−1^ bexarotene added daily. If treated early enough, a low dosage with an increased frequency of treatment could successfully remove Aβ burden, and then treatment frequency could slow enough to combat Aβ production.

## Supporting Information

S1 Text Note on *APP*/*PS1* mice. (TEX)

S2 Text Nondimensional analysis and sensitivity analysis. (PDF)

S1 Fig 90 day simulation of nine month-old *APP* / *PS1* mouse with 100 mg ⋅ kg^−1^ bexarotene treatment, healthy brain cells, diseased brain cells, and Aβ_42_. (EPS)

S2 Fig 90 day simulation of nine month-old *APP* / *PS1* mouse with 100 mg ⋅ kg^−1^ bexarotene treatment, apoE. (EPS)

S3 Fig Seven day simulation of 11-month-old *APP* / *PS1* mouse with mg ⋅ kg^−1^ bexarotene treatment. (EPS)

S4 Fig Contour plot of nondimensionalized system, 90 day simulation of nine month-old *APP*/*PS1* mouse with no treatment. *c*_0_ = 6.730, *c*_1_ = 6.057 ⋅ 10^−1^ and *T* = 6.729 ⋅ 10.
(EPS)

S5 Fig Contour plot of nondimensionalized system, 90 day simulation of nine month-old *APP*/*PS1* mouse with 100 mg ⋅ kg^−1^ bexarotene treatment. *c*_0_ = 6.730, *c*_1_ = 6.057 ⋅ 10^−1^ and *T* = 6.729 ⋅ 10.
(EPS)

S1 Table Solver run times and percent changes in final diseased neuron concentration and Aβ_42_ load. Each percent change is given in absolute value when compared to results from a run solved with ode45 for a 90 day simulation of a nine month-old *APP*/*PS1* transgenic mouse with mg ⋅ kg^−1^ bexarotene treatment.
(TEX)

S2 Table Percent change of the concentration of healthy brain cells in a 90 day simulation of nine-month-old *APP*/*PS1* mouse with 100 mg ⋅ kg^−1^ bexarotene treatment. Parameters are increased by 10%, and the approximate corresponding percent change of the system is given for 0 mg ⋅ kg^−1^ and 100 mg ⋅ kg^−1^ of bexarotene.
(TEX)
