# Correction: Early Treatment Critical: Bexarotene Reduces Amyloid-Beta Burden In Silico

**DOI:** 10.1371/journal.pone.0156474

**Published:** 2016-05-24

**Authors:** Joseph Rosenthal, Georges Belfort, David Isaacson

S1 Text, S1 and S2 Tables are incorrect. Please view the corrected S1 Text, S1 and S2 Tables here.

## Supporting Information

S1 Text Note on *APP*/*PS1* mice. (PDF)

S1 Table Solver run times and percent changes in final diseased neuron concentration and Aβ_42_ load. Each percent change is given in absolute value when compared to results from a run solved with ode45 for a 90 day simulation of a nine month-old *APP*/*PS1* transgenic mouse with mg ⋅ kg^−1^ bexarotene treatment.
(PDF)

S2 Table Percent change of the concentration of healthy brain cells in a 90 day simulation of nine-month-old *APP*/*PS1* mouse with 100 mg ⋅ kg^−1^ bexarotene treatment. Parameters are increased by 10%, and the approximate corresponding percent change of the system is given for 0 mg ⋅ kg^−1^ and 100 mg ⋅ kg^−1^ of bexarotene.
(PDF)
