# Supplementary material for: Early Treatment Critical: Bexarotene Reduces Amyloid-Beta Burden In Silico
Source: PLoS One. 2016 Apr 13;11(4):e0153150. doi: 10.1371/journal.pone.0153150 (PMC4830620; doi:10.1371/journal.pone.0153150)
Supplement: S2 Text — (PDF) [file pone.0153150.s006.pdf]

S2 Text. Nondimensional analysis and sensitivity analysis. In order to nondimensionalize the system for further analysis, consider the substitutions $t = T\tau$, $N(t) = \hat{N}n(\tau)$, $N_d(t) = \hat{N}n_d(\tau)$, $A(t) = \hat{A}a(\tau)$, $P_0(t) = \hat{P}_0 p_0(\tau)$, and $B(t) = \hat{B}b(\tau)$ where τ, n, n_d, a, and p_0 are dimensionless.

When substituting the above equations, eqs. (19) to (22) reduce to the following system:

$$\frac{dn}{d\tau} = -\left(\frac{\lambda_d \hat{A}a}{\hat{A}a + \alpha_0}\right)Tn + \left(\lambda_d - \frac{\lambda_d \hat{A}a}{\hat{A}a + \alpha_0}\right)Tn_d, \tag{1}$$

$$\frac{dn_d}{d\tau} = \left(\frac{\lambda_d \hat{A}a}{\hat{A}a + \alpha_0}\right)Tn - \left(\lambda_d - \frac{\lambda_d \hat{A}a}{\hat{A}a + \alpha_0}\right)Tn_d - \mu_d T n_d, \tag{2}$$

$$\frac{da}{d\tau} = \left(\frac{k_A \hat{N}^2 T}{\hat{A}}\right)\left(n_d^2 + n_d n\right) - k_{P_0} T \hat{P}_0 a p_0, \tag{3}$$

$$\frac{dp_0}{d\tau} = \left(\frac{k_R \rho_R \hat{B} b_T}{\hat{P}_0}\right)\hat{N}Tn - k_{P_0} T \hat{A} a p_0. \tag{4}$$

If $T = {\lambda_d}^{-1}$, $\hat{N} = \sqrt{\lambda_d \alpha_0 {k_A}^{-1}}$, $\hat{A} = \alpha_0$, $\hat{P}_0 = \lambda_d (k_{P_0})^{-1}$, and $\hat{B} = \lambda_d \hat{P}_0 (k_B \hat{N})^{-1}$ then eqs. (1) to (4) simplify to

$$\frac{dn}{d\tau} = -\frac{an}{a+1} + \left(1 - \frac{a}{a+1}\right)n_d, \tag{5}$$

$$\frac{dn_d}{d\tau} = \frac{an}{a+1} - \left(1 - \frac{a}{a+1}\right)n_d - c_0 n_d, \tag{6}$$

$$\frac{da}{d\tau} = n_d^2 + n_d n - a p_0, \tag{7}$$

$$\frac{dp_0}{d\tau} = b_T n - c_1 a p_0, \tag{8}$$

where $c_0 = \mu_d \left(\lambda_d\right)^{-1}$, and $c_1 = k_{P_0} \alpha_0 \left(\lambda_d\right)^{-1}$.

With this nondimensionalization, the model is now reduced to a system of three parameters: the timescale, T, and c_0 and c_1. The system can now be studied in a simplified form.

S1 Table. Index of values of nondimensionalized system parameters and scaling factors

Variable	Definition	Value
c_0	$\mu_{n_d}{\lambda_d}^{-1}$	$8.2 \cdot 10^{-2}$
c_1	$k_{P_0}\alpha_d{\lambda_d}^{-1}$	12.2623
T	${\lambda_d}^{-1}$	16.3934 days
$\hat{N}$	$\sqrt{\lambda_d \alpha_0 {k_A}^{-1}}$	4.1231 vol.$^{-1}$
$\hat{A}$	α_0	17 day^{-1}
$\hat{P}_0$	$\lambda_d {k_{P_0}}^{-1}$	1.3864 vol.$^{-1}$
$\hat{B}$	$\lambda_d \hat{P}_0 (k_R \rho_R \hat{N})^{-1}$	$1.74 \cdot 10^{-2}$ mg $\cdot$ kg^{-1}

With the nondimensionalized system given in eqs. (5) to (8), we can investigate the behavior of the system in neighborhoods about c_0 and c_1 over a 90 day period, thus T is fixed.

Before we investigate the sensitivity of the system to the parameters, we define the following differences:

$$\Delta N = N(t_f) - N(t_0) = \hat{N}\left(n(\tau_f) - n(\tau_0)\right), \tag{9}$$

$$\Delta N_d = N_d(t_f) - N_d(t_0) = \hat{N}\left(n_d(\tau_f) - n_d(\tau_0)\right), \tag{10}$$

$$\Delta A = A(t_f) - A(t_0) = \hat{A}\left(a(\tau_f) - a(\tau_0)\right), \tag{11}$$

$$\Delta P_0 = P_0(t_f) - P_0(t_0) = \hat{P}_0\left(p_0(\tau_f) - p_0(\tau_0)\right), \tag{12}$$

where t_f=90 days, and t_0=0 days. Changes with respect to c_0 and c_1 will be computed in the nondimensional system and then scaled to be dimensionful. Note that both Fig 4 and Fig 5 are centered about our calculated values of c_0 and c_1 given in Table 1.

Without treatment, there is no ApoE is introduced to the system, and thus the system is invariant to changes in c_1. It can be seen in Fig 4 that changes in c_0 do not significantly alter the behavior of the system.

With bexarotene added to the simulation, Fig 5 shows that c_0 and c_1 do not cause significant variation in ΔN. Furthermore, while c_1 has an influence on ΔN_d, it is influenced more heavily by c_0. With the introduction of bexarotene, the behavior of ΔA has changed and is now dominated by changes in c_0, as is ΔP_0.
