# Supplementary material for: Correction: Early Treatment Critical: Bexarotene Reduces Amyloid-Beta Burden In Silico
Source: PLoS One. 2016 May 24;11(5):e0156474. doi: 10.1371/journal.pone.0156474 (PMC4878765; doi:10.1371/journal.pone.0156474)
Supplement: S1 Text — (PDF) [file pone.0156474.s001.pdf]

S1 Text. Note on *APP*/*PS1* mice. Trinchese and Liu reported formic acid-extractable $A\beta_{42}$ levels in mice expressing both the Swedish mutation of *APP* (K670N:M671L) and human *PS1* (M146L) [1]. These mice present a similar pathology to the *APP*/*PS1* mice used by Cramer et al., which express both the Swedish mutation of *APP* and the $\Delta E9$ mutation of *PS1* [2, 3].
