# Supplementary material for: Correction: Early Treatment Critical: Bexarotene Reduces Amyloid-Beta Burden In Silico
Source: PLoS One. 2016 May 24;11(5):e0156474. doi: 10.1371/journal.pone.0156474 (PMC4878765; doi:10.1371/journal.pone.0156474)
Supplement: S1 Table — Each percent change is given in absolute value when compared to results from a run solved with ode45 for a 90 day simulation of a nine month-old APP/PS1 transgenic mouse with mg ⋅ kg−1 bexarotene treatment. (PDF) [file pone.0156474.s002.pdf]

S1 Table. Solver run times and percent changes in final diseased neuron concentration and $A\beta_{42}$ load. Each percent change is given in absolute value when compared to results from a run solved with `ode45` for a 90 day simulation of a nine month-old *APP*/*PS1* transgenic mouse with 100 $mg \cdot kg^{-1}$ bexarotene treatment

Solver	Time (s)	Percent change in final concentration	
		Diseased neuron	$A\beta_{42}$ load
`ode45`	2.660	—	—
`ode23`	2.496	0	$2.157 \cdot 10^{-3}$
`ode23s`	3.241	$2.077 \cdot 10^{-2}$	$2.372 \cdot 10^{-2}$
`ode23t`	1.980	$1.246 \cdot 10^{-2}$	$2.372 \cdot 10^{-2}$
`ode23tb`	2.659	0	$2.372 \cdot 10^{-2}$
`ode113`	4.930	0	$2.372 \cdot 10^{-2}$
