# Supplementary material for: Correction: Early Treatment Critical: Bexarotene Reduces Amyloid-Beta Burden In Silico
Source: PLoS One. 2016 May 24;11(5):e0156474. doi: 10.1371/journal.pone.0156474 (PMC4878765; doi:10.1371/journal.pone.0156474)
Supplement: S2 Table — Parameters are increased by 10%, and the approximate corresponding percent change of the system is given for 0 mg ⋅ kg−1 and 100 mg ⋅ kg−1 of bexarotene. (PDF) [file pone.0156474.s003.pdf]

S2 Table. Percent change of the concentration of healthy brain cells in a 90 day simulation of nine-month-old *APP/PS1* mouse with 100 $\text{mg} \cdot \text{kg}^{-1}$ bexarotene treatment. Parameters are increased by 10%, and the approximate corresponding percent change of the system is given for 0 $\text{mg} \cdot \text{kg}^{-1}$ and 100 $\text{mg} \cdot \text{kg}^{-1}$ of bexarotene.

Parameter changed	Percent change of healthy brain cells	
	0 $\text{mg} \cdot \text{kg}^{-1}$ bex	100 $\text{mg} \cdot \text{kg}^{-1}$ bex
λ_d	14	18
α_d	3.3	5.6
μ_d	2.6	$5.4 \cdot 10^{-1}$
k_A	11	18
k_{P_0}	0	$2.8 \cdot 10^{-2}$
k_B	0	4.3
